# Molecular screening of mosquitoes for filarioid helminths in Slovenia

**DOI:** 10.1186/s13071-026-07295-3

**Published:** 2026-05-04

**Authors:** Urška Glinšek Biškup, Nataša Knap, Miša Korva, Tatjana Avšič Županc, Katja Adam, Tea Knapič, Samo Zakotnik, Barbara Šoba

**Affiliations:** 1 Institute of Microbiology and Immunology, Faculty of Medicine, University of Ljubljana, Ljubljana, Slovenia; 2 Department of Biodiversity, Faculty of Mathematics, Natural Sciences and Information Technologies, University of Primorska, Koper, Slovenia; 3 Slovenian Museum of Natural History, Ljubljana, Slovenia

**Keywords:** *Dirofilaria repens*, *Setaria tundra*, *Setaria labiatopapillosa*, Slovenia, Xenoonitoring, Mosquito, Vector

## Abstract

**Background:**

*Dirofilaria repens* infections have already been reported in both dogs and humans in Slovenia. Xenomonitoring of mosquitoes for filarioid helminths has been used in many studies across Europe to analyze the autochthonous occurrence of filarioids. The aim of this study was to identify potential mosquito vectors of *D. repens* and other filarioid helminths in Slovenia, to shed light on the significance and extent of possible autochthonous transmission of filarioids in the country.

**Methods:**

This study was a part of the Slovenian nationwide screening program of mosquitoes for filarioid helminths and viruses. Adult mosquitoes were collected from numerous sites throughout Slovenia in 2021 and 2022, and were screened for filarioid helminths using real-time polymerase chain reaction (PCR) targeting a 94-base pair (bp) fragment of the 12S rRNA gene from the mitochondrial genome. Positive samples, which were confirmed by conventional PCR targeting 667 bp of the COI gene, were sequenced and compared with GenBank sequences for species identification.

**Results:**

Almost 56,000 adult mosquitoes were screened for filarioid helminths, which were grouped into 5446 pools. The number of mosquitoes in each pool ranged from 1 to 60. Of all the screened pools, 29 were positive for filarioid helminths, giving a total minimum infection rate (MIR) of 0.52 per 1000 mosquitoes. Alongside *D. repens*, which was detected in six pools, the following were also identified: *Setaria tundra* in 15 pools; *S. labiatopapillosa* in two pools; and an unknown filarioid species in six mosquito pools. In the study, we found eight mosquito species in Slovenia that may transmit filarioid worms, including *Aedes vexans*, *Ae. albopictus*, *Ae. cinereus*, *Ae. sticticus*, *Anopheles maculipennis* s.l., *An. claviger*, *Coquillettidia richiardii*, and *Culex pipiens* s.l., indicating that multiple species could contribute to the local transmission of these parasites.

**Conclusions:**

The detection of filarioid helminths in Slovenian mosquitoes is consistent with the previous findings of potential mosquito species that can carry *D. repens*, *S. tundra*, and *S. labiatopapillosa* in Europe, indicating the potential for local transmission. Consequently, dirofilarioid infections in dogs and humans, and setarial infections in roe deer, should be considered. Further research is required to better understand the epidemiology of these infections in Europe.

**Graphical Abstract:**

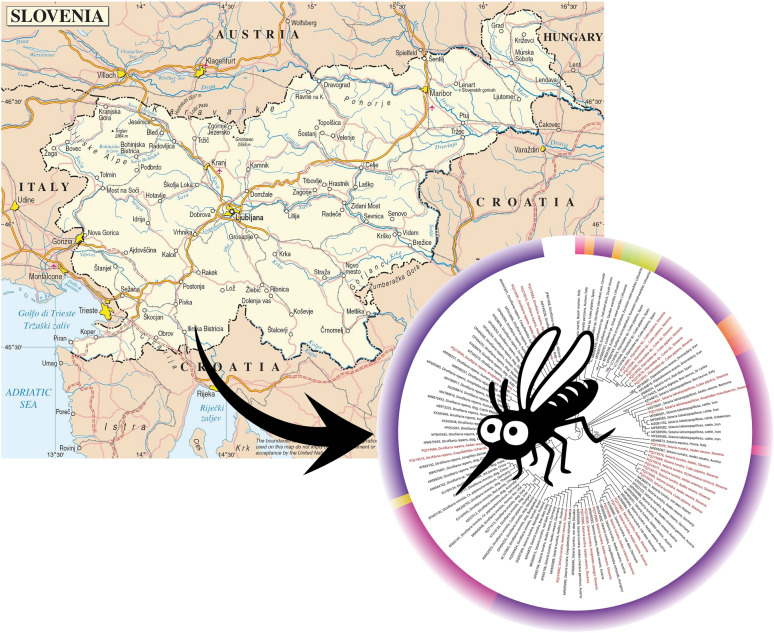

## Background

Several mosquito species, primarily from the genera *Culex*, *Aedes*, and *Anopheles*, are capable of transmitting mosquito-borne filarioids, such as *Dirofilaria* spp. and *Setaria* spp. [1–3]. In the Mediterranean region, *Culex pipiens* s.l., *Anopheles maculipennis* s.l., and *Aedes albopictus* are identified as potential vectors of filarioid helminths [2, 4, 5]. Some species, such as *Cx. pipiens* s.l. and *An. maculipennis* s.l., are widely distributed throughout Europe, while others, such as *Ae. albopictus*, are expanding their range northward from established populations in the Mediterranean [6].

*Dirofilaria immitis* and *D. repens* are the most important filarioid helminths of veterinary and/or medical relevance in Europe. They are widespread in tropical, subtropical, and temperate regions [3]. *Dirofilaria immitis* causes a severe disease (heartworm disease) in dogs and other carnivores and occasionally infects humans, while *D. repens* usually causes a nonpathogenic subcutaneous infection in dogs and is the main cause of human dirofilariasis in the Old World [3]. *Dirofilaria immitis* and *D. repens* are endemic in southern Europe, where the number of human cases is increasing, and are spreading to northern Europe [7]. *Dirofilaria immitis*, commonly known as canine heartworm, is a parasitic nematode primarily affecting dogs but occasionally infecting humans as an incidental host. Human infections are rare and usually asymptomatic, but in some cases, the larvae migrate to the pulmonary arteries and cause respiratory symptoms such as cough and chest pain [3, 8, 9].

*Dirofilaria repens* in humans does not usually reach the adult stage, but remains in an immature form. It can cause *larva migrans* syndrome and form subcutaneous nodules. The worm often reaches the ocular region and occasionally other organs, such as the lungs [7].

Setariae are generally considered nonpathogenic in their natural hosts. However, emerging severe outbreaks of *S. tundra*-associated peritonitis and perihepatitis in reindeer and moose in Finland have been reported, causing significant economic losses [10, 11]. *Setaria tundra* lives in the abdominal cavity of cervids. The main vertebrate hosts of *S. tundra* are reindeer and roe deer [12]. Intermediate hosts are various mosquito species, mainly *Aedes* spp. [13]. Human infections have not been described. *Setaria tundra* has been reported in 15 European countries so far. In recent years, its range has expanded by thousands of kilometers, extending from the subpolar to the subtropical climatic zone. Since 1969, this parasite has been found in Austria, Bulgaria, Switzerland, Sweden, Norway, Finland, and Germany. After 2000, it was also found in Italy, France, Poland, Slovakia, Hungary, Spain, Croatia, and Denmark [11, 14]. Its presence in Belarus, Czech Republic, Slovenia, and Estonia is very likely [15]. *Setaria labiatopapillosa* normally colonizes the peritoneal cavity of cattle without apparent associated pathology [1]. It can become pathogenic in unusual mammalian hosts and is associated with central nervous system lesions leading to nonspecific neurological disease [1]. The only confirmed cases of human infection have been reported in Bucharest (south-eastern Romania), where four patients living in the same area presented with ocular infections [16]. *Aedes vexans* appears to be one of the most efficient natural vectors for this species [1].

The present study was part of the Slovenian nationwide screening of mosquitoes for various pathogens such as viruses and filarioid helminths. *Dirofilaria repens* infections have already been reported in dogs and humans in Slovenia [17–20]. However, owing to the proximity to neighboring countries of Croatia and Italy, both endemic for dirofilariasis, it is difficult to determine whether there is also autochthonous transmission of dirofilariasis in Slovenia. The aim of this study was to identify potential mosquito vectors of *D. repens* and other filarioid helminths in Slovenia by molecular screening to shed light on the significance and extent of possible autochthonous transmission of filarioids in the country.

## Methods

### Mosquito collection and DNA extraction

As part of a nationwide screening for filarioid helminths in Slovenia, adult mosquitoes were collected at numerous sites throughout Slovenia in 2021 and 2022. We sampled mosquitoes at 226 locations to ensure surveillance of a significant part of the country.

Mosquitoes were sampled using three types of traps: Centers for Disease Control and Prevention (CDC) light miniature traps, BG-Sentinel traps baited with CO_2_, and gravid traps. The traps operated for 24 h to capture both diurnal and nocturnal mosquito species. Traps were set in rural and urban areas near water sources or near animals.

Captured mosquitoes were collected, put on dry ice in the field, and maintained under cold conditions throughout the testing process. The majority of the mosquitoes were identified to species level using morphological identification on the basis of anatomical features as our primary identification method. If this was not sufficient, cytochrome oxidase I (COI) barcoding was carried out to identify the species [21, 22].

The collected mosquitoes, which were screened for the presence of DNA of filarioid helminths, were counted and grouped into pools with of up to 60 mosquitoes per pool (from minimum of 1 to maximum of 60) by species, sex, collection site, and date. A total of 34,477 and 21,218 mosquitoes grouped into 2981 and 2465 pools were screened for filarioid DNA in 2021 and 2022, respectively.

The whole mosquitoes were used for the analysis. Each pool of mosquitoes was homogenized in 600 µL of Roswell Park Memorial Institute (RPMI) medium using Tissue Lyser (Retsch for Qiagen, Hilden, Germany). Nucleic acid was extracted from the 200 µL of mosquito homogenate with the BioRobot EZ1-XL Advanced (Qiagen, Germany) using the EZ1 Virus Mini Kit version 2.0 (Qiagen, Germany) and eluted in 60 µL. The remaining homogenate was stored for further analysis.

### Molecular detection of filarioid helminths and sequence analysis

The presence of DNA of filarioid helminths was detected by real-time polymerase chain reaction (PCR) targeting 94 base pairs (bp) of the 12S rRNA gene from the mitochondrial genome using the primers FILA-F, FILA-R, and probe FILA-P, as previously described [23]. The real-time PCR was performed with Platinum^®^ Quantitative PCR SuperMix-UDG with ROX (Invitrogen, Thermo Fisher Scientific, USA) according to the manufacturer’s protocol on QuantStudio 5 Real-Time PCR System (Applied Biosystems, Thermo Fisher Scientific, USA).

Real-time PCR-positive samples were confirmed by conventional PCR targeting 667 bp of COI gene using primers COIintF and COIintR [24] and sequencing for species discrimination of filarioid helminths. Conventional PCR was performed with HotStarTaq DNA Polymerase (Qiagen, Germany) according to the manufacturer’s protocol on Applied Biosystems 2720 Thermal Cycler (Applied Biosystems, Thermo Fisher Scientific, USA). PCR products were subsequently purified and bidirectionally sequenced using BigDye terminator chemistry on an ABI PRISM 310 genetic analyzer (Applied Biosystems, Thermo Fisher Scientific, USA). Finally, all sequences generated were assembled and corrected using CLC Main Workbench 7.9.1 (Qiagen, Germany) and then compared with sequences available in GenBank using the Basic Local Alignment Search Tool (BLAST) to confirm species identity.

### Phylogenetic analysis and infection rate calculation

The generated sequences were then used for phylogenetic analysis. First, the new sequences were aligned with selected sequences from the literature using MAFFT [25] (version 7.526). The alignment was then used for the phylogenetic analysis with IQ-Tree [26, 27] (version 2.2.0). The best fitting model was selected using Model Finder as part of IQ-Tree and was GTR + F + I + R4. The phylogenetic tree was drawn using TVBOT [28] (version 2.6.1).

A Microsoft Excel add-in (Program PooledInfRate, version 3.0) developed by Brad Biggerstaff (CDC, Fort Collins, CO, USA) was used to calculate the infection rates from pooled data.

## Results

Almost 56,000 adult mosquitoes representing six genera were subjected to the screening for filarioid helminths, with autochthonous *Aedes* (64.8%), *Culex* (19%) and invasive *Aedes* (9.5%) being the most frequent mosquito genera examined (Table 1). The group of invasive *Aedes* spp. included the species *Ae. albopictus*, *Ae. japonicus*, and *Ae. koreicus* and the group of autochthonous *Aedes* spp. included species *Ae. vexans*, *Ae. cinereus*, and *Ae. sticticus*.

**Table 1:** Mosquito species and pools examined

Mosquito species	No. of mosquitoes screened (%)		No. of pools screened/positive for filarioid DNA	
	Year 2021	Year 2022	Year 2021	Year 2022
Autochthonous *Aedes* spp.^a^	24,570 (71.3)	11,536 (54.37)	1188/13	851/2
Invasive *Aedes* spp.^b^	2081 (6.0)	3187 (15.0)	416/0	427/1
*Anopheles* sp.	1197 (3.5)	1138 (5.4)	324/0	282/3
*Coquillettidia* sp.	623 (1.8)	335 (1.6)	96/1	86/0
*Culex* sp.	5834 (16.9)	4930 (23.2)	871/3	768/6
*Culiseta* sp.	166 (0.5)	87 (0.4)	81/0	47/0
*Uranotaenia* sp.	6 (0.02)	0 (0)	5/0	0/0
Unknown	0 (0)	5 (0.02)	0/0	4/0
Total	34,477	21,218	2981/17	2465/12

^a^Including *Ae. vexans*, *Ae. cinereus*, and*Ae. sticticus*

^b^Including *Ae. albopictus*, *Ae. japonicus*, and *Ae. koreicus*

Out of 5446 pools examined, 29 (0.5%) were confirmed to contain filarioid DNA (17/2981 (0.6%) in 2021 and 12/2465 (0.5%) in 2022 (Table 1) by the conventional PCR targeting COI gene. The DNA of *D. repens* has been detected in 6 pools, *S. tundra* in 15 pools, and *S. labiatopapillosa* in 2 pools. In addition, six of the obtained sequences could not be assigned to a species owing to insufficient identities to GenBank entries (Table 2).

**Table 2:** Details of mosquito pools positive for filarioid DNA, including species identification and collection data

Filarioid species	GenBank accession no.	Maximum percentage (%) identity to GenBank entry (accession no.)^a^	Collection site (region)	Collection date	Mosquito species	Pool size (no. of mosquitoes)
*S. tundra*	PQ219580	99.8% (e.g., MF695092)	Lower Sava	15 June 2021	*Ae. vexans*	5
*D. repens*	PQ219583	100% (e.g., PP465883)	Lower Sava	15 June 2021	*Ae. vexans*	1
*D. repens*	PQ219584	100% (e.g., PP552047)	Drava	17 June 2021	*Ae. vexans*	8
*S. tundra*	PQ219585	100% (e.g., MK360914)	Mura	29 June 2021	*Ae. vexans*	7
*S. tundra*	PQ219586	100% (e.g., MK360915)	Mura	29 June 2021	*Ae. vexans*	7
*S. tundra*	PQ219587	100% (e.g., MF695095)	Drava	17 June 2021	Mixture of *Aedes* spp.^b^	4
*S. tundra*	PQ219588	100% (e.g., MF695095)	Gorizia	22 June 2021	*Ae. vexans*	2
*S. tundra*	PQ219589	100% (e.g., MK360914)	South-eastern Slovenia	8 July 2021	*Cx. pipiens* s.l	3
*D. repens*	PQ219573	100% (e.g., PP465883)	Lower Sava	13 July 2021	*Ae. vexans*	1
*S. tundra*	PQ219574	100% (e.g., MK360914)	Drava	15 July 2021	*Ae. sticticus*	5
*D. repens*	PQ219575	100% (e.g., KX265048)	Drava	15 July 2021	*Cq. richiardii*	19
*S. tundra*	PQ219576	100% (e.g., MK360914)	Mura	29 July 2021	Mixture of *Aedes* spp.^b^	50
*S. tundra*	PQ219577	100% (e.g., MK360914)	Savinja	5 August 2021	*Ae. cinereus*	2
*S. tundra*	PQ219578	100% (e.g., MK360914)	Littoral–Inner Carniola	13 August 2021	*Ae. vexans*	4
Filarioidea	PQ219579	98.5% (e.g., LC107818)	Carinthia	20 August 2021	*Culex* sp.	1
Filarioidea	PQ219581	99.5% (e.g., LC107818)	Gorizia	3 Sept. 2021	*Cx. pipiens* s.l	1
*S. tundra*	PQ219582	99.9% (e.g., MK360915)	Mura	5 Oct. 2021	*Ae. vexans*	42
Filarioidea	PQ219596	99.7% (e.g., LC107818)	Gorizia	8 June 2022	*Cx. pipiens* s.l	2
Filarioidea	PQ219597	98.5% (e.g., LC107818)	Gorizia	8 June 2022	*Cx. pipiens* s.l	4
*S. tundra*	PQ219598	100% (e.g., MF695095)	Savinja	23 June 2022	*Ae. cinereus*	2
*S. tundra*	PQ219599	99.8% (e.g., MK360914)	Savinja	29 June 2022	*Ae. vexans*	10
*S. tundra*	PQ219600	100% (e.g., MK360915)	Drava	14 June 2022	*An. claviger*	2
*S. labiatopapillosa*	PQ219601	99.7% (e.g., MF589582)	Gorizia	5 July 2022	*Cx. pipiens* s.l	8
Filarioidea	PQ219590	99% (e.g., LC107818)	Gorizia	20 July 2022	*Cx. pipiens* s.l	1
*D. repens*	PQ219591	100% (e.g., PP465883)	Coastal–Karst	15 July 2022	*Ae. albopictus*	40
*S. labiatopapillosa*	PQ219592	99.8% (e.g., MF589582)	Central Slovenia	4 August 2022	*An. maculipennis* s.l	7
*S. tundra*	PQ219593	100% (e.g., MK360914)	South-eastern Slovenia	9 August 2022	*Cx. pipiens* s.l	1
*D. repens*	PQ219594	100% (e.g., PP465883)	Mura	11 August 2022	*An. maculipennis* s.l	14
Filarioidea	PQ219595	98.8% (e.g., LC107818)	Central Slovenia	25 August 2022	*Culex* sp.	7

^a^Analysis of maximum identity to GenBank entries was performed on 26 August 2024

^b^Including *Ae. vexans*, *Ae. cinereus*, and *Ae. sticticus*

The overall minimum infection rate (MIR) per 1000 mosquitoes was calculated at 0.52 (0.49 in 2021 and 0.56 in 2022) using the PooledInfRate Excel add-in, which employs a maximum likelihood estimation method that accounts for variable pool sizes. MIR differed among mosquito genera, the highest values of MIR occurred in the species *Anopheles* sp., *Coquillettidia* sp., *Culex* sp., and autochthonous *Aedes* spp. and were estimated as 1.29, 1.04, 0.84, and 0.42, respectively (Table 2, 3).

**Table 3:** Mosquito species collected with information on the number of screened mosquito specimens, tested pools, screening results and MIRs per 1000 mosquito specimens

Mosquito species	Number of individual mosquitoes		Number of pools	Number of positive pools	Minimum infection rate (MIR)
Autochthonous *Aedes* spp.^a^	36,106		2039	15	0.42
Invasive *Aedes* spp.^b^	5268		843	1	0.19
*Anopheles* sp.	2335		606	3	1.29
*Coquillettidia* sp.	958		182	1	1.04
*Culex* sp.	10,764		1639	9	0.84
*Culiseta* sp.	253		128	0	0.00
*Uranotaenia* sp.	6		5	0	0.00
Unknown sp.	5		4	0	0.00

^a^Including *Ae. vexans*, *Ae. cinereus*, and*Ae. sticticus*

^b^Including *Ae. albopictus*, *Ae. japonicus*, and *Ae. koreicus*

The *D. repens*-positive pools were composed of three *Ae. vexans* pools and one pool each of *Cq. richiardii*, *Ae. albopictus*, and *An. maculipennis* s.l. specimens. Seven *Ae. vexans*, two *Ae. cinereus*, a mixture of *Aedes* spp. and *Cx. pipiens* s.l., and one *Ae. sticticus* and *An. claviger* pool contained *S. tundra* DNA. The two *S. labiatopapillosa*-positive pools were *Cx. pipiens* s.l. and *An. maculipennis* s.l. pools. The DNA of unknown filarioid species was detected in six pools of *Cx. pipiens* s.l. (Table 2).

The COI DNA sequences of the identified filarioids found in the mosquitoes have been deposited in GenBank under accession nos. PQ219573–PQ219601.

BLAST analysis of all *D. repens* COI sequences obtained revealed a 100% nucleotide identity to those in GenBank. In contrast, *S. labiatopapillosa* and *S. tundra* COI sequences showed an identity range of 99.7–99.8% and 99.8–100% to the sequences available in GenBank, respectively. Six positive samples not assigned to a species owing to insufficient identities to GeneBank entries, displayed a homology of 98.5–99.7% in direct comparison (Table 2).

**Fig. 1:**
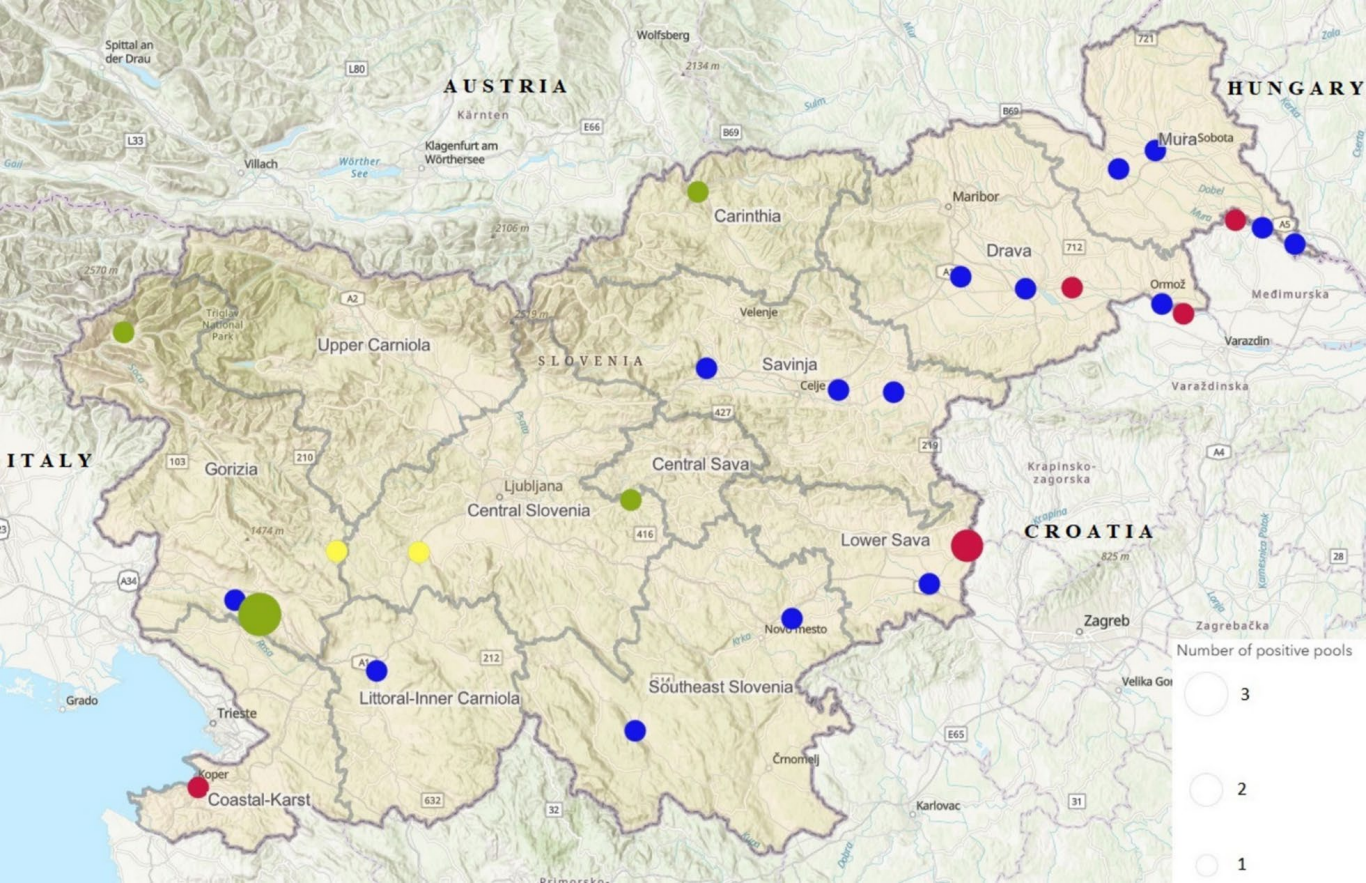
Geographic origin of the mosquitoes tested positive for filaroid helminths (red dots: *D. repens*, blue dots: *S. tundra*, yellow dots: *S. labiatopapillosa*, green dots: Filarioidea—unknown filarioid species)

All filariae-carrying mosquitoes were collected between mid-June and early September in 2021 and 2022 in 10 out of 12 Slovenian regions. Six of the positive mosquito pools were collected in the Gorizia region, followed by the Drava and Mura regions with five positive pools each (Table 2, Fig. 1).

Mosquitoes collected from four different regions (two pools from the Lower Sava, two from the Drava, one each from the Mura and the Coastal–Karst region) tested positive for *D. repens*. All of the *D. repens*-positive mosquito pools were from regions bordering Croatia. Additionally, two of these positive pools were from regions also close to Hungary and one close to Italy (Fig. 1).

*Setaria tundra* was found in mosquito pools from seven different regions in Slovenia. Four positive pools were from the Mura region, three each from the Drava and Savinja regions, two from the south-eastern Slovenia region, and one each from the Lower Sava, Gorizia, and Coastal–Karst region.

*Setaria labiatopapillosa* was detected in one mosquito pool from the Gorizia region, and one from the Central Slovenia region.

Most of the unknown filarioid species were detected in mosquitoes from the Gorizia region, bordering Italy (four pools), the Carinthia, bordering Austria (one pool), and the Central Slovenia region (one pool) (Table 2, Fig. 1).

**Fig. 2:**
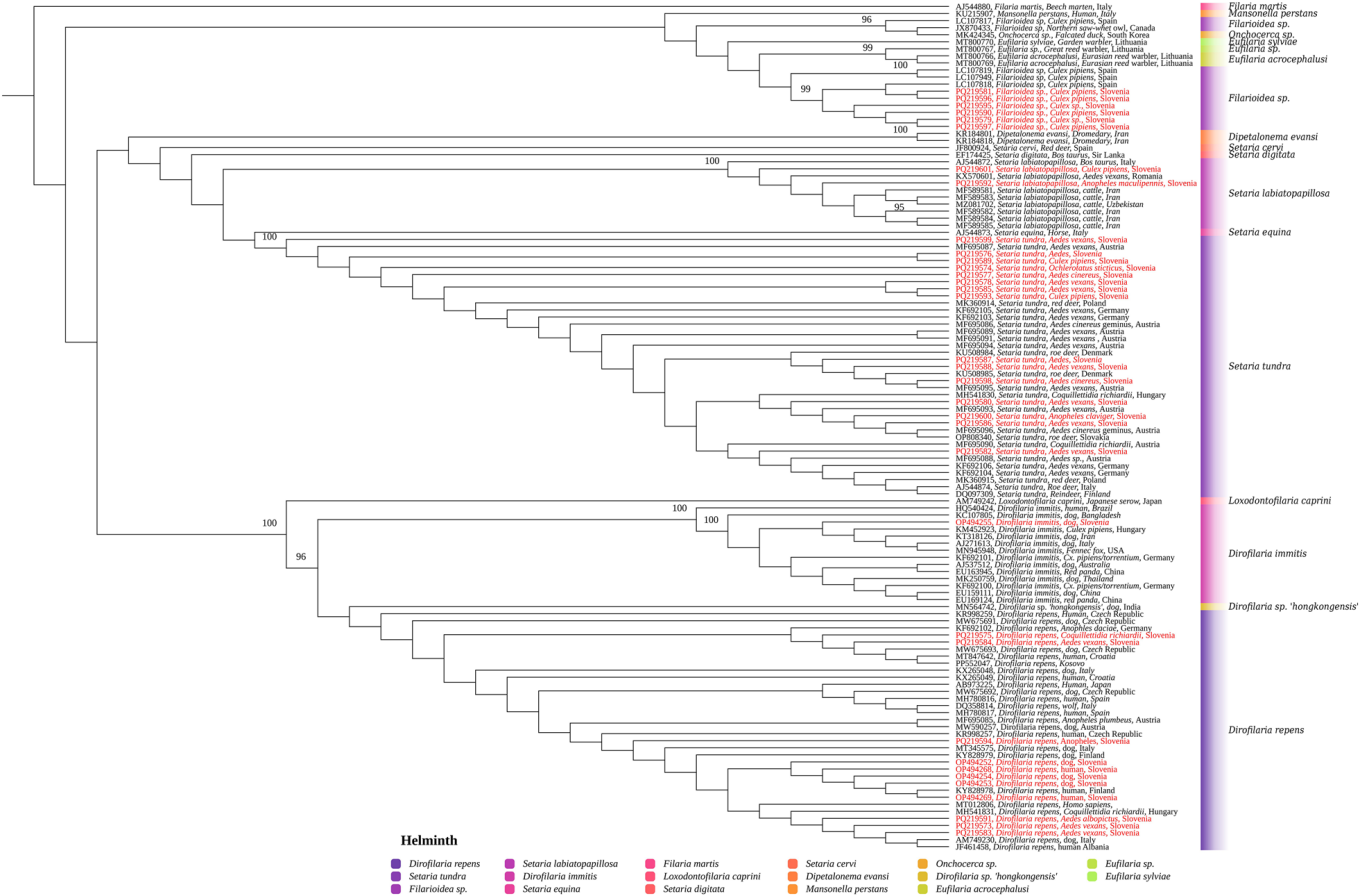
Phylogenetical analysis of newly obtained filarioid cytochrome oxidases sequences and selected sequences from the literature. The phylogenetic tree was constructed with IQ-Tree using GTR + F + I + R4 model. Newly obtained sequences are marked with red, and the ultrafast bootstrap support > 95 is marked on the branches

Phylogenetic analyses confirmed the molecular identification by clustering all obtained sequences of *D. repens*, *S. tundra*, and *S. labiatopapillosa* into their respective species clades, each supported by high bootstrap values. According to the phylogenetic, tree *D. repens* sequences obtained clustered with other *D. repens* sequences from Europe. Similarly, *S. tundra* sequences from our study clustered with related *S. tundra* sequences from countries across the Europe. However, *S. labiatopapillosa* sequences obtained were close to sequences from Italy, Romania, and Iran. The unknown filarioid DNA sequences clustered with related sequences from Spain (Fig. 2).

## Discussion

Dirofilariasis has been historically endemic in the Mediterranean European countries [7]. However, epidemiological studies and recent clinical reports indicate a significant increase in local infections of dogs and humans with *D. immitis* and/or *D. repens* in central and northern Europe during the last two decades. Climatic changes and the facilitation of pet travel seem to have contributed to this expansion, with the major factor likely being the rate of undiagnosed dogs that continue to perpetuate the parasite life cycle. In particular, there has been a marked increase in *D. repens* infections in animals and humans in central and north-eastern Europe [3, 7, 9, 17, 29–33]. *Dirofilaria repens* has been already detected in dogs and humans in Slovenia [17–20], but this is the first study on the vector mosquito species for filarioid parasites in Slovenia, confirming autochthonous transmission of *D. repens* in the country.

During this study, mosquito species, especially from the genera *Aedes* and *Culex*, were trapped and screened in large numbers; these genera have previously been identified as potential vectors of filarioid parasites [1, 2].

In many studies, xenomonitoring of mosquitoes for filarioid helminths has been used to analyze the autochthonous occurrence of *Dirofilaria* spp. in a specific geographical area [23, 32, 34–44]. The effectiveness of molecular xenomonitoring relies on adequate sampling of the vector population, accurate determination of their infection status, and linking the presence of the pathogen in the vector to human or animal infections. To assess the risk of disease transmission to vertebrates correctly, it is crucial to identify vector species in an area and estimate their abundance and distribution [13]. Inter-study comparisons could be difficult owing to the differences in the xenomonitoring results obtained with different PCR assays.

In the present study, we tested for the presence of filarioid DNA in 55,695 mosquitoes from seven different genera, sampled in 12 Slovenian regions and grouped into 5446 pools. We used real-time PCR [23] as screening method and conventional PCR in combination with sequencing [24] for confirmation and filarioid species identification. Filarioid DNA was confirmed with conventional PCR and sequencing in 29 pools, giving a total MIR of 0.52 per 1000 mosquitoes. While we used only one confirmatory PCR, we used the MIR instead of the estimated infection rate (EIR), as it was suggested by Masny et al. [43] and defined in a xenomonitoring study in Slovakia [35]. Regarding mosquito species, the highest MIR in our study was detected in *Anopheles* sp. (MIR = 1.29), followed by *Coquillettidia* sp. (MIR = 1.04) and *Culex* sp. (MIR = 0.84). Among invasive *Aedes* spp., only one pool was positive for filarioid species (MIR = 0.19), while among autochthonous *Aedes* spp., there were 15 positive pools (MIR = 0,42). Although the percentage of positive mosquitoes was low, this study allows us to gain insight into species that could be vectors for filarioid species.

We found *D. repens* DNA in 6/29 positive pools of mosquitoes, including species of *Ae. vexans*, *Cq. richiardii*, *Ae. albopictus*, and *An. maculipennis* s.l., suggesting that these species could serve as potential vectors of *D. repens* in Slovenia. All these species have already been identified as potential vectors of *D. repens* throughout Europe [32, 35–37, 40–42, 45, 46].

All *D. repens*-positive mosquito pools were collected in regions where Slovenia borders neighboring countries, mainly close to Croatia and also to Italy and Hungary (Fig. 1), suggesting a possible introduction of *D. repens* from these countries. The prevalence of *D. repens* infection in dogs in Croatia is up to 47.3% [33] and up to 30% in Italy and Hungary, where *D. repens* has also been detected in mosquitoes [7, 47].

In Slovenia, infections with *D. repens* have been diagnosed in dogs and humans in the Central, Coastal–Karst, south-eastern, and north-eastern regions of the country. Autochthonous cases of *D. repens* infection have been suspected in dogs and humans with no travel history to endemic countries in Costal–Karst region, north-eastern and central Slovenia [17, 19, 20]. One *D. repens*-positive mosquito pool from the north-eastern part of Slovenia was phylogenetically close to strains from dogs and humans (Fig. 2), from the same part of Slovenia, which indicates autochthonous transmission of *D. repens* in this region [17]. A *D. repens*-positive mosquito pool was also detected in the Costal–Karst region, where, as already mentioned, an autochthonous human case has also been diagnosed. Moreover, it was found in one pool of *Ae. albopictus*, which belongs to the group of invasive *Aedes* spp. [19].

The *S. tundra*-positive mosquito pools accounted for more than half of all detected positive pools (15/29); most *S. tundra*-positive pools were *Ae. vexans* pools, followed by *Cx. pipiens* s.l., *Ae. cinereus*, *Ae. sticticus*, and *An. claviger* pools. In European countries, *S. tundra* has already been detected in all these mosquito species [13, 23, 37, 38, 40, 43, 48]. Moreover, *Ae. vexans* has been confirmed as a predominant species of mosquitoes infected with *S. tundra* [13, 23, 37, 40, 48]. *Setaria tundra* is endemic in the countries where roe deer are present. Its presence has already been reported in all of Slovenia’s neighboring countries: Austria, Hungary, Croatia, and Italy [15]. Its presence in Slovenia confirms the risk of this parasite spreading to other European countries [15].

Some of the *S. tundra* strains identified in our study, which were found in mosquitoes collected in regions near the borders with Austria and Hungary, were phylogenetically close to the strains found in these countries. The similarity was also observed with strains from Italy, Germany, Poland, and Denmark, suggesting the spread of *S. tundra* from these countries (Fig. 2).

In our study, *S. labiatopapillosa* was detected in one *Cx. pipiens* s.l. and one *An. maculipennis* s.l. pool. *S. labiatopapillosa* has also been found in *An. maculipennis* s.l. mosquitoes in the study of Azari-Hemidian et al. [49] from Iran, while to our knowledge, it has not yet been found in *Cx. pipiens* s.l. mosquitoes. In the study by Ionica et al. [44]*, S. labiatopapillosa* has been found in *Ae. vexans*, which has also been identified as an efficient natural vector of this filarioid species in the study by Cancrini et al. [1]. This filarioid species typically lives in the peritoneal cavity of cattle without causing noticeable health issues [1], which may explain the limited epidemiological data available. When this filarioid species infects atypical hosts such as sheep or horses, it can become pathogenic and cause lesions in the central nervous system, leading to nonspecific neurological conditions [1]. Given the zoonotic nature of the parasite, further research is needed to investigate its presence in definitive hosts and potential vectors.

The *S. labiatopapillosa* strains from our study were phylogenetically close to strains from Italy and Romania (Fig. 2). One of them was found in the Gorizia region, close to the Italian border, and was phylogenetically close to *S. labiatopapillosa* strain AJ544872 found in cattle from Italy, and one in the Central Slovenia region.

As in other published studies, unknown filarioid species detected in 6/29 mosquito-positive pools were found exclusively in *Culex* sp. [23, 37, 40, 48, 50]. *Culex pipiens* s.l. has a feeding preference for birds [51]; therefore, the unknown filarioid species could be an avian nematode species, as discussed by Übleis et al. [48], Kronefeld et al. [37], and Czajka et al. [23]. A BLAST search of the GenBank database for sequences most similar to ours revealed the highest nucleotide sequence identity (93%) with avian Onchocercidae parasites, e.g., *Eufilaria acrocephalusi* and *E. sylviae*. The sequences were phylogenetically close to strains from Spain and Lithuania (Fig. 2). This study confirms the widespread distribution of these undefined parasites in Europe. Further studies, including investigations of potential vertebrate hosts and detailed morphological identification, are required to determine the origin of these unidentified parasites.

*Dirofilaria immitis* was not detected in the present study. Cases of *D. immitis* infections in dogs and humans have been reported from all Slovenia neighboring countries—Croatia, Italy, Austria, and Hungary [33, 52–54]. Furthermore, a confirmed autochthonous case in a cat has been reported in Austria [55]. This pathogen has also been confirmed in mosquitoes in Hungary and Italy [4, 34, 39, 56].

## Conclusions

It can be concluded that *D. repens*, *S. tundra*, *S. labilopapilosa*, and other unknown filarioid species are present in Slovenia. Further studies are needed to monitor in more detail the situation of *D. repens* and *D. immitis* in Slovenia and neighboring countries. Globalization and climate change increase the risk of spreading of *D. repens* from southern Europe and *Setaria* spp. from northern regions. The presence of vector-competent mosquitoes and suitable climatic conditions in these areas could support the development of these parasites. Consequently, the potential for dirofilarioid infections in dogs and humans, as well as setarial infections in roe deer, should be considered. The epidemiology of these infections in Europe requires further research.

## Data Availability

The data supporting the findings of this study are available within the paper.

## Abbreviations

COI: Cytochrome c oxidase subunit I
s.l.: Sensu lato (in the broad sense)
